# Assessing morbidity, mortality, and survival in patients with peritoneal carcinomatosis undergoing cytoreductive surgery and hyperthermic intraperitoneal chemotherapy

**DOI:** 10.1590/0100-6991e-20233421-en

**Published:** 2023-03-31

**Authors:** JAIRO SEBASTIÁN ASTUDILLO VALLEJO, FABIO LOPES DE QUEIROZ, ANTÔNIO LACERDA, PAULO ROCHA FRANÇA, BRENO XAIA MARTINS DA COSTA, RODRIGO ALMEIDA PAIVA, SILVÉRIO LEONARDO MACEDO GARCIA, SERGIO BOTREL SILVA

**Affiliations:** 1 - Hospital Felicio Rocho, Coloproctologia - Belo Horizonte - MG - Brasil; 2 - Hospital Felicio Rocho, Cirurgia Geral - Belo Horizonte - MG - Brasil; 3 - Hospital Felicio Rocho, Anestesiologia - Belo Horizonte - MG - Brasil

**Keywords:** Mortality, Survival, Peritoneal Diseases, Cytoreduction Surgical Procedures, Hyperthermic Intraperitoneal Chemotherapy, Neoplasias Peritoneais, Sobrevida, Quimioterapia Intraperitoneal Hipertérmica, Procedimentos Cirúrgicos de Citorredução, Mortalidade

## Abstract

Peritoneal carcinomatosis (PC) indicates advanced stage cancer, which is generally associated with a poor outcome and a 6 to 12 months. Cytoreductive surgery (CRS) with hyperthermic intraperitoneal chemotherapy (HIPEC) is an option for treating patients with primary PC, such as mesothelioma, or secondary PC, such as colorectal cancer (CRC) or pseudomixoma. Until recently, such patients were deemed untreatable.

**Objective:**

The purpose of this study was to assess the results of CRS + HIPEC in patients with PC. Postoperative complications, mortality and survival rates were evaluated according to the diagnosis.

**Results:**

Fifty-six patients with PC, undergoing full CRS + HIPEC between October 2004 and January 2020, were enrolled. The mortality rate was 3.8% and the morbidity rate was 61.5%. Complications were significantly higher in proportion to the duration of surgery (p<0.001). The overall survival rates, as shown in the Kaplan-Meyer curve, were respectively 81%, 74% and 53% at 12, 24 and 60 months. Survival rates according to each diagnosis for the same periods were 87%, 82% and 47% in patients with pseudomixoma, and 77%, 72% and 57% in patients with CRC (log-rank 0.371, p=0.543).

**Conclusion:**

CRS with HIPEC is an option for pacients with primary or secondary PC. Although complication rates are high, a longer survival rate may be attained compared to those seen in previously published results; in some cases, patients may even be cured.

## RESUMO

O diagnóstico de carcinomatose peritoneal (CP), indica um estágio avançado do câncer e em geral está associado a um mau prognóstico com sobrevida média variando de 6 a 12 meses. A cirurgia citorredutora (CRS) associada à quimioterapia intraperitoneal hipertérmica (HIPEC) tem sido descrita como uma opção de tratamento para os pacientes portadores de CP primária como nos portadores de mesotelioma, ou secundária como em portadores de cancer colorretal ou pseudomixoma, até recentemente considerados sem possibilidade terapêutica.

**Objetivo:**

Avaliar os resultados do tratamento de pacientes portadores de CP submetidos a CRS + HIPEC. Foram analisadas as taxas complicações pós-operatórias, mortalidade e a sobrevida desses pacientes.

**Resultados:**

Foram incluídos 56 pacientes com CP, submetidos a c CRS + HIPEC, no período de Outubro 2004 a Janeiro 2020. A taxa de mortalidade foi de 3,8% e taxa de morbidade de 61,5%, sendo a ocorrência de complicações significativamente maior quanto maior o tempo cirúrgico (p<0,001). A sobrevida global pela curva de Kaplan-Meyer foi de 81%, 74% e 53% em 12, 24 e 60 meses respectivamente. Já a sobrevida por diagnóstico nos mesmos períodos foi de 87%, 82%, 47% para os pacientes portadores de pseudomixoma e de 77%, 72% e 57% para pacientes portadores de câncer colorretal (Log -RANK 0,371, p=0,543).

**Conclusão:**

A CRS + HIPEC é uma opção de tratamento para pacientes portadores de CP primária ou secundária. Embora com taxa de complicações elevadas, pode proporcionar aumento da sobrevida quando comparado com resultados prévios da literatura e em alguns casos até a cura.

## INTRODUCTION

The diagnosis of peritoneal carcinomatosis (PC), whether primary or secondary, indicates cancer in advanced stage and is associated with a poor prognosis. In the European EVOCAPE I multicenter study, the median survival rates were 6.9 and 6.5 months for colorectal and gastric cancer associated with peritoneal carcinomatosis, respectively. The average survival rate in patients with advanced stage ovarian cancer ranges from 12 to 23 months, and in those with malignant peritoneal mesothelioma, most studies report survival rates of less than one year^1^. In 1989, Chu et al. demonstrated the results of 100 patients with PC of different origins, which were 45 colorectal, 20 pancreas, six gastric, four small intestine, two appendix, two unknown primary, and 21 miscellaneous. Median survival was six months for colorectal origin, 0.7 months for pancreatic origin, and one month for gastric origin^2^. In 2002, Jayne et al. performed a retrospective analysis of 349 patients with PC from 3,019 patients with colorectal cancer, observing that the median survival was only seven months, being affected by the extent of the PC and the stage of the primary tumor^3^. In 2015, a Swedish study concluded that cytoreductive surgery (CRS) plus hyperthermic intraperitoneal chemotherapy (HIPEC) may be superior to systemic treatment with Oxaliplatin for patients with CRC and resectable isolated peritoneal metastases (median overall survival 25 months vs 18 months, p=0.04)^4^. Kusamura et al. also showed better overall survival results in patients with Pseudomyxoma treated with CRS plus HIPEC versus patients treated with CRS alone^5^.

CRS associated with HIPEC has been described as a treatment option for patients with PC, until recently considered beyond therapeutic possibility^6^.

The use of cytoreductive surgery was introduced by JV Meigs for the treatment of advanced ovarian cancer with peritoneal metastases in the 1930s. The development of this aggressive surgical approach gained greater acceptance in the scientific community in the 60s and 70s, with positive treatment results of peritoneal Pseudomyxoma and metastatic ovarian tumors^7^. The development of this technique for the treatment of some peritoneum diseases advanced until the 20^th^ century and was optimized by Sugarbaker, who proposed six specific procedures for removal of the peritoneum, in order to achieve complete cytoreduction^8^. On the other hand, in the 1970s, other studies showed the possible benefits of using chemotherapy applied directly to the peritoneum via the abdominal route to control coelomic implants^9^. Hyperthermia was introduced by Spratt et al. in the 1980s^10^ ^-^ ^12^. Later Zimm et al. and Howell et al., in phase I and II studies, showed benefits of this treatment, and CRS with HIPEC became the standard treatment for patients with Pseudomyxoma and peritoneal mesothelioma^13^ ^,^ ^14^.

A natural corollary of these results was the use of this treatment for peritoneal metastases from other primary sites, such as the stomach, pancreas, sarcomas, and colon. Although in some of these diseases the initial results were poor, in peritoneal disease from colorectal cancer the results were promising. Elias et al., for example, reported a 5-year survival of up to 50% in patients with peritoneal carcinomatosis who underwent CRS and HIPEC with intrabdominal Oxaliplatin and concomitant venous 5-FU^15^. Recently, the Prodige 7 study did not show survival benefits among patients in the group that underwent cytoreductive surgery alone compared with those in the group that underwent cytoreduction and HIPEC^16^. Wisselink et al., however, question the results of this study, emphasizing a short time of exposure to the drug, as well as the effectiveness of the drug that was used, reinforcing that the results obtained with the use of Oxaliplatin could not be extrapolated to the results of Mitomycin, another drug used in the HIPEC^17^.

Over the years, peritoneal carcinomatosis treatment centers have been established in the United States, Europe, Japan, and Brazil. The feasibility, efficacy, and safety of CRS and HIPEC have been proven in several clinical trials^18^ ^,^ ^19^.

Despite the success of previous initial results, there are still many controversies regarding the use of CRS + HIPEC and few prospective controlled studies on the subject. In addition, the characteristics of patients undergoing this type of treatment are very heterogeneous, as it most often consists of a rescue treatment, used when previous less aggressive surgeries or systemic oncological treatment have failed, which makes it difficult to compare different studies. The lack of quality clinical studies or even consistent retrospective data makes it necessary to publish results from different services and regions of the world, as well as in different types of tumors, so that more definitive conclusions can be drawn about the benefits of this treatment. Thus, the present study aims to evaluate the results obtained by the Center for the Treatment of Peritoneal Diseases at Felício Rocho Hospital, as well as the mortality, morbidity, and survival rates of patients with peritoneal carcinomatosis of different sites, treated with CRS and HIPEC.

## METHODS

This is a retrospective, observational study, carried out at the Felício Rocho Hospital, from October 2004 to January 2020. The study was previously approved by the institution’s Ethics Committee. We studied 56 patients whom underwent complete CRS with HIPEC, all operated on by the same surgeon after training in the technique.

We included patients with peritoneal carcinomatosis secondary to colorectal cancer and peritoneal Pseudomyxoma, as well as some cases of patients with rare tumors, such as sarcoma and mesothelioma, and patients with gastric and ovarian tumors who had undergone previous surgical and chemotherapy treatments and had peritoneal recurrence.

Most patients with PC of colorectal origin had already undergone surgical treatment with previous colectomies. In the case of patients with PC due to Pseudomyxoma and ovarian tumor, most had undergone previous laparotomy and laparoscopy for the diagnosis of peritoneal carcinomatosis and were subsequently referred to our peritoneal disease treatment service.

All patients underwent computed tomography of the chest, abdomen, and pelvis or nuclear magnetic resonance (NMR) of the abdomen and pelvis to assess the extent of the disease and rule out distant metastases. In the case of dubious lesions, such as pulmonary, bone, or retroperitoneal lymph node metastases, which could contraindicate the procedure, positron emission tomography (PET-CT) was performed and, when present, the patients were not submitted to the CRS and HIPEC procedure. The presence of resectable liver metastases was not an isolated criterion to contraindicate the procedure if it they could be resected.

Patients considered fit for surgery underwent wide laparotomy and had their peritoneal carcinomatosis index (PCI) evaluated^20^. After laparotomy, patients with colorectal cancer with a PCI of up to 24 and those with Pseudomyxoma, mesothelioma, or ovarian cancer and a PCI of up to 39 were considered suitable for cytoreductive surgery. After calculating the PCI, the Complete Cytoreduction (CC) procedure was performed, which was evaluated using the CC score, in which a CC-0 score indicates that there is no visible tumor after cytoreduction, a CC-1 score indicates tumor nodules <2.5mm that persist after debulking; a CC-2 score indicates tumor nodules between 2.5mm and 2.5cm; and a CC-3 score indicates tumor nodules >2.5cm or a confluence of unresectable tumor nodules at any site. We reseted affected organs when necessary, and peritoneal implants separately when indicated. Peritoneal segments affected by implants were resected according to the procedures described by Sugarbaker^7^. After complete cytoreduction, patients underwent hyperthermic intraperitoneal chemotherapy with temperature between 40°C and 42°C. The drug schemes used for intraperitoneal chemotherapy were Mitomycin C (35mg/m^2^) for 90 minutes^17^, Oxaliplatin (460mg/m^2^) intrabdominally for 30 minutes, associated with intravenous 5-FU (20mg/m^2^) administered 30 minutes before abdominal perfusion^17^ or Cisplatin for 90 minutes^17^ ^,^ ^21^, used in 4 cases. In most patients, we used the open “Coliseum” technique^18^, and in some cases, the closed technique. After QT, the cavity was revised and irrigation with 0.9% saline solution was performed. Then, the abdomen was closed, and the drains used for QT were left in situ and removed on the following days when drainage was less than 100ml in 24 hours. Intensive care unit admission was not mandatory, being jointly decided by the anesthesiology and surgery teams^20^ ^-^ ^21^.

To evaluate the overall results of the service in the treatment of patients with peritoneal carcinomatosis, we analyzed the demographic data of the patients, such as sex, age, peritoneal carcinomatosis index, duration of surgery, length of stay in intensive care, hospital stay, and the occurrence of complications according to the Clavien-Dindo classification^22^ ^,^ ^23^. In patients with colon tumor and peritoneal Pseudomyxoma, we performed the analysis of the results separately according to the type of primary tumor. In patients with sarcoma, mesothelioma, ovarian tumor, and gastric tumor, a detailed analysis of the results was not possible due to the small sample of each of these tumor types. We evaluated overall survival rate, disease-free survival, and compared survival within each group according to the type of primary tumor and the drug used in HIPEC.

### Statistical analysis

We present quantitative data by the descriptive measures mean, median, standard deviation, and percentiles, and categorical data by absolute and relative frequencies. We tested quantitative data for normality using the Kolmogorov-Smirnov test. We used the ANOVA test to compare means and the Dunnet’s T3 test for multiple comparisons. We estimated the overall and disease-free survival curves using the Kaplan-Meier method and compared the overall survival curves using the Log-Rank test. For all tests, we adopted a significance level of 5%, and used the SPSS version 23.0 Software. 

## RESULTS

We evaluated 56 patients with peritoneal carcinomatosis who underwent complete CRS and HIPEC, from October 2004 to January 2020, at the Center for the Treatment of Peritoneal Diseases at the Felício Rocho Hospital. All patients had peritoneal carcinomatosis and CC0 surgery was performed. Thirty-six patients (64%) were female, 24 patients (42%) were diagnosed with Pseudomyxoma, 19 (33%) with CRC and 13 (23%) had other diseases: one gastric cancer, seven ovary tumors, two mesotheliomas, one sarcoma, and one neuroendocrine tumor (Table 1).

**Table 1:** Distribution of patients by sex and diagnosis (n=56).

Variables	n	%
Sex		
Female	36	64.3
Male	20	35.7
Diagnosis		
CRC	19	33.9
Pseudomyxoma	24	42.9
Others	13	23.2

The patients’ average age was 51.1 years, with an average hospital stay of 16.9 days, and mean eight days spent in the ICU. The mean duration of surgery was 7.9 hours, ranging from one to 16 (Table 2).

**Table 2:** Distribution of patients undergoing CRS + HIPEC, according to age, length of stay, and duration of surgery (n=56).

Variables	n*	Average	Standard deviation	Minimum	Maximum	Median	Percentiles		
							25	75
Age	56	51.1	14.3	14.0	84.0	52.5	43.3	60.5
Surgery (hours)	47	7.9	3.4	1.0	16.0	7.0	5.3	10.2
HIPEC time	52	74.3	23.0	30.0	90.0	90.0	60.0	90.0
ICU stay (days)	55	8.0	8.7	1.0	37.0	4.0	2.0	10.0
Hospital stay (days)	54	16.9	12.0	4.0	53.0	12.0	8.0	23.0

*number; HIPEC: intraperitoneal hyperthermic chemotherapy; ICU: intensive care unit.

The mean peritoneal carcinomatosis index of all patients was 15, 11 for CRC, 18 for Pseudomyxoma, and 14 for other diseases (Table 3).

**Table 3:** Peritoneal Carcinomatosis Index by Diagnosis (n=56).

PCI	n*	Average	Standard Deviation	Standard Error	95% confidence interval		Minimum	Maximum
					Lower limit	Upper limit		
CRC	19	11.16	5.805	1,332	8.36	13.96	2	24
Pseudomyxoma	24	18.50	12,752	2,603	13.12	23.88	0	39
OTHERS	13	14.38	6,923	1920	10.20	18.57	3	27
Total	56	15.05	10.001	1,336	12.38	17.73	0	39

*number; PCI: peritoneal carcinomatosis index; CRC: colorectal cancer.

There were complications in 32 patients (61.5%), of which 23 were severe (Clavien-Dindo type IV). The mortality rate was 3.8% (Table 4). The occurrence of complications was significantly higher the longer the surgical time (p<0.001) (Table 5).

**Table 4:** Occurrence of complications according to Clavien-Dindo classification.

Variables	n*	%
Complications		
None	20	38.5
I	3	5.8
II	3	5.8
III	1	1.9
IV	23	44.2
V	2	3.8

*number.

**Table 5:** Frequency of complications in patients undergoing CRS + HIPEC, according to surgical time.

Surgery time (hours) and Complications	n	Average	Standard deviation	95% CI		Minimum	Maximum	p-value *	Multiple Comparisons	p-value **
None	18	5.9	2.1	4.9	6.9	1.0	10.0	<0.001	N vs. I and II	0.681
I and II	5	6.7	1.4	5.0	8.4	5.0	8.0		N vs. III and IV	<0.001
III, IV, and V	22	9.8	3.6	8.2	11.4	4.0	16.0		I and II vs. III and IV	0.17

*ANOVA test; **Dunnet’s T3 test.

The main complications, from mild to more severe, classified according to Clavien-Dindo were surgical wound infection, urinary tract infection, systemic inflammatory response syndrome (SIRS), sepsis, pneumothorax, and death.

When analyzing the two groups of patients with CRC and Pseudomyxoma, which are the most frequent pathologies, we found that the complication rate for patients with CRC was 63%, and for the ones with Pseudomyxoma group, 50%, SIRS being the most frequent complication.

There was a trend towards a higher rate of complications in patients who used Mitomycin, regardless of the type of tumor (p=0.078), type IV (severe) complications occurring in 19 (50%) of 38 patients, with two (5%) deaths. On the other hand, serious complications occurred in five (36%) of 14 patients who used Oxaliplatin (Table 6). 

**Table 6:** Clavien-Dindo complications according to the type of chemotherapy used, Mitomycin versus Oxaliplatin, in patients undergoing CRS + HIPEC (p=0.078).

Variables	None	I	II	III	IV	V	Total
Mitomycin Mean PCI 16.6	12 (31%)	2 (5%)	2 (5%)	1 (2,5%)	19 (50%)	2 (5%)	38
Oxaliplatin Mean PCI 12.7	8 (57%)	1 (7%)	-	-	5 (36%)	-	14

**Graph 1:**
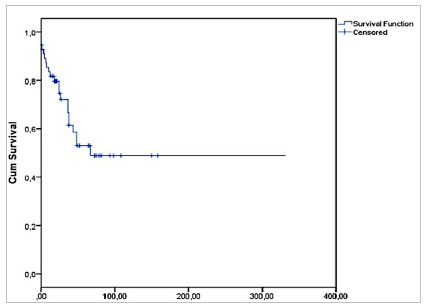
Overall survival of patients undergoing CRS + HIPEC.

**Graph 2:**
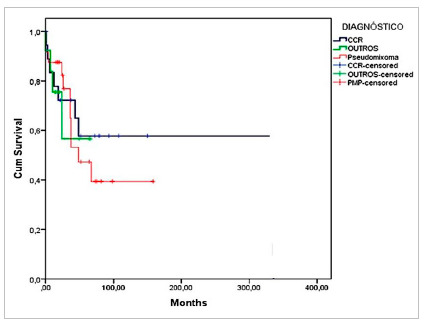
Survival of patients undergoing CRS + HIPEC according to the diagnoses of CRC and Pseudomyxoma (Log-RANK 0.371, p=0.543).

Overall survival according to the Kaplan-Meyer curve was 81% at 12 months, 74 % at 24 months, and 53% at five years (Graph 1). Survival by diagnosis in the same periods was 87%, 82%, and 47% for patients with Pseudomyxoma and 77%, 72%, and 57% for patients with colorectal cancer (Log-RANK 0.371, p=0.543) (Graph 2).

As for the survival of patients with other diagnoses, it was lower, of 56% in 24 months.

**Graph 3 ch3:**
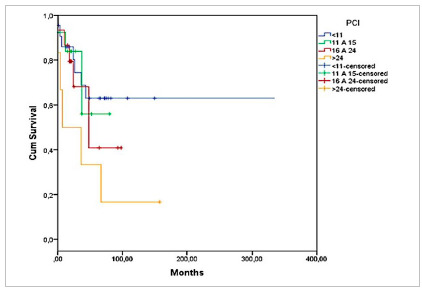
Overall survival of patients undergoing CRS + HIPEC according to PCI (n=56).

**Graph 4:**
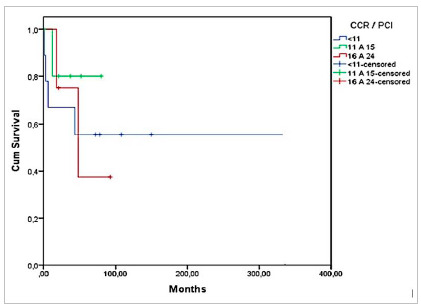
Survival of patients with CRC undergoing CRS + HIPEC according to PCI (p=0.733, n=19).

**Graph 5:**
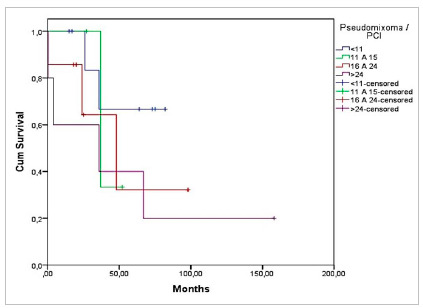
Survival of patients with Pseudomyxoma undergoing CRS + HIPEC according to PCI (p=0.447, n=24).

Graph 3 shows the overall survival according to PCI, and Graphs 4 and 5, the survival according to diagnosis and PCI. There was greater survival in patients with CRC in the group with PCI 11-15, of 80% in 12 months, but without significant difference (p=0.733). In patients with Pseudomyxoma, survival was higher in those with PCI <11, 83% at 24 months, but also without significant difference (p=0.447) (Graph 5).

[Fig ch3]

**Graph 6 ch6:**
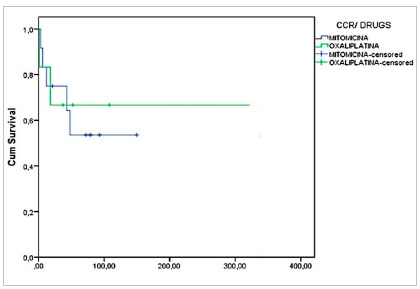
Survival according to diagnosis (CRC) and type of drug used for HIPEC (p=0.831, n=19).

**Graph 7:**
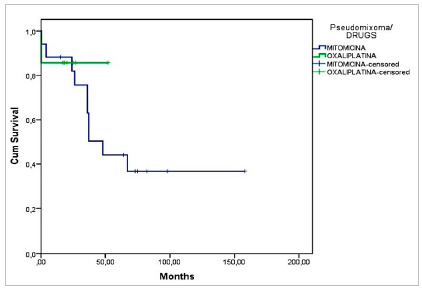
Survival according to diagnosis (Pseudomyxoma) and type of drug used for HIPEC (p=0.601, n=24).

Graph 6 depicts the survival of patients with CRC undergoing CRS and HIPEC according to the chemotherapy used. Patients with CRC treated with Oxaliplatin displayed a survival of 73% in 60 months, and those treated with Mitomycin, 55%, without a statistically significant difference (p=0.831). There was also no significant difference in survival at five years in patients with Pseudomyxoma treated with Oxaliplatin when compared with those who received Mitomycin (80% x 50%, p=0.601) (Graph 7).

[Fig ch6]

**Graph 8:**
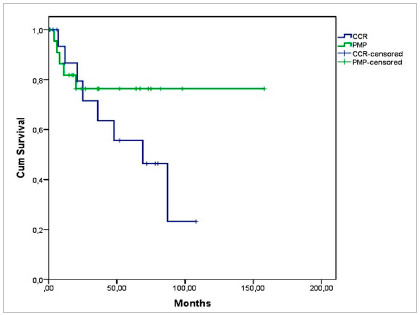
Disease Free Survival according to diagnosis - CRC and Pseudomyxoma - (p=0.248, n=43).

Disease-free survival was 86% at 12 months and 55% at 60 months in patients with CRC, and 81% at 12 months and 76% at 60 months in patients with Pseudomyxoma (p=0.248) (Graph 8).

## DISCUSSION

Primary or secondary peritoneal carcinomatosis indicates an advanced stage of the disease. Systemic treatment presents disappointing results and the use of cytoreductive surgery and HIPEC provides better results, with increased overall and disease-free survival, although with high morbidity and mortality rates, due to extensive surgeries, often performed in patients already debilitated by oncological treatments or prior surgeries. Therefore, it is very important that the services that perform CRS + HIPEC periodically analyze their overall morbidity and mortality results, which are much more due to the surgical trauma itself, associated with the effects of hyperthermic chemotherapy, the experience of the multidisciplinary team, and the indication of treatment, than to the type of primary tumor. It is also important to observe long-term survival results, even in less frequent tumors such as sarcomas, mesothelioma, and ovarian tumors, to guide the indication of this therapeutic modality in these types of neoplasia.

Our study showed that the overall survival of patients with PC undergoing CRS and HIPEC was 81% at 12 months, 74% at 24 months, and 66% at 36 months, superior to those reported in the European study EVOCAPE I and in historical series in the literature that performed only cytoreductive surgery^5^ ^,^ ^24^.

The overall survival results in patients with Pseudomyxoma were 87% at 12 months, 82% at 24 months, and 47% at five years, confirming the results reported by Sugarbaker, as well as by two Mayo Clinic publications from 1990^24^, which reported a 10-year survival of 32% for low-grade Pseudomyxoma and a five-year survival of 6% for adenocarcinoma of the appendix that underwent CRS alone, reaffirming the role of CRS plus HIPEC in the treatment of this type of pathology.

In patients with peritoneal carcinomatosis from colorectal cancer, overall survival was 77% at 12 months, 72% at 24 months, and 57% at five years. Elias et al. also reported that the survival of patients with CRC with PC treated with CRS and HIPEC can reach up to 50% in five years. Recently, the Prodige 7 study found worse overall survival results; moreover, it did not demonstrate survival benefits among patients in the group that underwent cytoreductive surgery alone compared with those in the group undergoing cytoreduction + HIPEC^16^. Wisselink et al., in another study on the subject, question the results of Prodige 7, emphasizing the short time of exposure to the drug during HIPEC, as well as the effectiveness of the used drug itself, reinforcing that the results obtained with the use of Oxaliplatin could not be extrapolated to results with Mitomycin, another drug used in HIPEC^17^. The data from our study, although lacking a control group, suggest that CRS + HIPEC provide better overall and disease-free survival results for patients with CRC when compared with historical series in the literature^1^ that only assessed cytoreduction or systemic chemotherapy, which have very reduced survival.

Another factor that may be important for treatment outcome is the patient having been previously exposed to the drug that will be used in HIPEC, which could generate resistance of the cells implanted in the peritoneum, thus reducing its effect. Wisselink et al. consider that performing CRS + HIPEC with Mitomycin is a better option for the treatment of patients with CRC, since the drug exposure time is longer, around 90 minutes, and in general the tumor cells of these patients have not yet been exposed to this drug, which is not used in the systemic treatment of CRC^17^. Other authors such as Glockzin et al. suggested that the use of Oxaliplatin for hyperthermic intraperitoneal chemotherapy in combination with intravenous 5-FU does not increase perioperative morbidity and may improve outcomes. They conclude that Oxaliplatin should be considered in standard protocols for HIPEC in patients with peritoneal carcinomatosis due to appendicular and colorectal adenocarcinoma^25^ ^,^ ^26^. Recently, the discontinuity of Mitomycin supply by the pharmaceutical industry hampered the use of this drug by hyperthermic intraperitoneal chemotherapy.

The results in our study, despite lacking statistically significant differences with respect to PCI, demonstrated, as well as others in the literature, that overall survival may be higher in patients with PCI <11, which would be expected due to the lower volume of disease^1^, and that results may even improve in patients with Pseudomyxoma when compared with CRC, probably because it is a less aggressive and more indolent disease. In the group of patients with CRC, those with PCI between 11-15 showed a tendency towards better survival results, which was also evidenced in the Prodige 7 study, in which, in the subgroup analysis, patients with PCI of 11-15 undergoing CRS + HIPEC had a median overall survival of 41.6 months versus 32.7 months in those who underwent surgery alone (p=0.0209)^16^, suggesting that a PCI >15 negatively impacts the survival of patients with CRC and those with a PCI lower than 11 may not benefit from HIPEC, or the benefit is not offset by the occurrence of complications and surgical mortality.

In the comparative analysis of data from these studies, there was no difference in overall survival according to the type of drug used (Oxaliplatin x Mitomycin) both for patients with Pseudomyxoma and for those with colorectal cancer. Zhang et al. reported that Oxaliplatin and Mitomycin could achieve comparable survival when used in HIPEC for carcinomatosis in CRC.

CRS + HIPEC has a high complication rate. In our study, the overall rate of complications according to the classification and Clavien-Dindo was 61.5%, with death in the first 30 days occurring in 3.8% of patients. In addition, the occurrence of complications was significantly higher the longer the surgical time.

The rate of complications associated with performing HIPEC with Oxaliplatin is higher. In the Prodige 7 study the rate of complications observed with the use of Oxaliplatin was high, around 42% within 30 days after surgery. Zhang et al., considering the higher incidence of complications associated with Oxaliplatin than with Mitomycin, deem the latter the safest to be adopted in clinical routines. In our study, the most used chemotherapy was Mitomycin, applied in 38 patients, versus 14 who received Oxaliplatin. Severe complications (Clavien-Dindo IV) occurred in 50% of the patients who received Mitomycin and in 36% of the patients who used Oxaliplatin (p=0.078), demonstrating a trend towards a higher rate of complications with Mitomycin, as opposed to the reported in the literature. These results could be explained by the greater number of patients receiving Mitomycin, as well as the higher PCI in this group, in addition to the small number of patients in the sample.

When comparing our study with the one published by Rabelo et al. in 2012 at the same hospital, we observed a significant increase in survival at 12 months, from 61% to 81%, and a decrease in the complications rate, from 83% to 61%. These data show that better results can be obtained with the increase of the experience of the multidisciplinary team and a better selection of patients that occurs with the improvement of the involved professionals^27^.

Patients with peritoneal carcinomatosis are in an advanced stage of the disease, with limited survival and few treatment options. The combination of CRS + HIPEC in the treatment of these patients may increase survival, possibly even in those with CRC, and in cases of Pseudomyxoma and mesothelioma, even curing the disease. There is no consensus among different authors about the ideal chemotherapy regimen to be used for HIPEC. Patients with a lower PCI had a longer survival, and among those with CRC, the greatest benefit was seen in the group with a PCI between 11 and 15. The mortality rate found was acceptable, but CRS + HIPEC entails a high morbidity rate, reinforcing the concept that these procedures should be a therapeutic option for very well selected patients and performed by a well-trained team with experience in the technique and in highly complex procedures. Even for patients with rare tumors such as sarcomas and mesothelioma with carcinomatosis and in those with carcinomatosis from gastric and ovarian tumors that did not respond adequately to conventional treatments, CRS + HIPEC can be a treatment option, with the intention of prolonging survival and reducing the occurrence of abdominal complications such as ascites and intestinal obstruction. In these cases, the indication must be individualized and shared with the patient and the oncologist.

Among the shortcomings of our study, we highlight the small sample size, the lack of a control group that did not receive HIPEC, and the fact that there was no randomization for the type of drug used, Oxaliplatin versus Mitomycin.

## CONCLUSION

More prospective, randomized, comparative studies are needed to determine whether or not the association of HIPEC with CRS is beneficial, especially in patients with CRC. There is also a need for further studies to define the best treatment regimen to be used, especially in terms of morbidity, mortality, and long-term oncological outcome in patients with peritoneal carcinomatosis.
